# A T Cell Suppressive Circuitry Mediated by CD39 and Regulated by ShcC/Rai Is Induced in Astrocytes by Encephalitogenic T Cells

**DOI:** 10.3389/fimmu.2019.01041

**Published:** 2019-05-10

**Authors:** Cristina Ulivieri, Domiziana De Tommaso, Francesca Finetti, Barbara Ortensi, Giuliana Pelicci, Mario Milco D'Elios, Clara Ballerini, Cosima T. Baldari

**Affiliations:** ^1^Department of Life Sciences, University of Siena, Siena, Italy; ^2^Department of Experimental Oncology, European Institute of Oncology, Milan, Italy; ^3^Department of Translational Medicine, Piemonte Orientale University “Amedeo Avogadro”, Novara, Italy; ^4^Department of Experimental and Clinical Medicine, University of Florence, Florence, Italy

**Keywords:** astrocyte, EAE, neuroinflammation, molecular adaptor, T cell signaling, ATP-degrading enzyme

## Abstract

Multiple sclerosis is an autoimmune disease caused by autoreactive immune cell infiltration into the central nervous system leading to inflammation, demyelination, and neuronal loss. While myelin-reactive Th1 and Th17 are centrally implicated in multiple sclerosis pathogenesis, the local CNS microenvironment, which is shaped by both infiltrated immune cells and central nervous system resident cells, has emerged a key player in disease onset and progression. We have recently demonstrated that ShcC/Rai is as a novel astrocytic adaptor whose loss in mice protects from experimental autoimmune encephalomyelitis. Here, we have explored the mechanisms that underlie the ability of Rai^−/−^ astrocytes to antagonize T cell-dependent neuroinflammation. We show that Rai deficiency enhances the ability of astrocytes to upregulate the expression and activity of the ectonucleotidase CD39, which catalyzes the conversion of extracellular ATP to the immunosuppressive metabolite adenosine, through both contact-dependent and–independent mechanisms. As a result, Rai-deficient astrocytes acquire an enhanced ability to suppress T-cell proliferation, which involves suppression of T cell receptor signaling and upregulation of the inhibitory receptor CTLA-4. Additionally, Rai-deficient astrocytes preferentially polarize to the neuroprotective A2 phenotype. These results identify a new mechanism, to which Rai contributes to a major extent, by which astrocytes modulate the pathogenic potential of autoreactive T cells.

## Introduction

Astrocytes are the first CNS resident cells encountered by infiltrating autoreactive T cells in multiple sclerosis (1, 2). Astrocytes contribute to neuroinflammation in multiple sclerosis and in the mouse experimental autoimmune encephalomyelitis (EAE) model by promoting encephalitogenic T-cell activation through their ability to act as antigen presenting cells (APC) and to upregulate the T-cell costimulatory molecules B7-1 and B7-2 (3–6). Intriguingly, T-cell suppression by astrocytes has also been documented, resulting from their ability to promote antigen-independent surface upregulation of inhibitory molecules on T cells, including the inhibitory receptor CTLA-4 and the ectonucleotidases CD39 and CD73 (7, 8). Additionally, astrocytes actively influence the generation and maintenance of effector T cells both by modulating CD4^+^ T-cell polarization to Th1 cells and by supporting IL-2-dependent Treg cell survival (9, 10). The finding that different subsets of reactive astrocytes are induced following CNS injury, of which the A1 is neurotoxic and the A2 neuroprotective (11), adds further complexity to the role of astrocytes in CNS diseases.

Astrocytes are themselves targets of infiltrating autoreactive T cells. Antigen-independent, contact-dependent upregulation of the integrin ligands VCAM-1 and ICAM-1 on astrocytes cocultured with activated T cells has been reported (8). Additionally, infiltrating Th1 and Th17 cells modulate astrocyte function via contact-independent mechanisms involving the release of inflammatory mediators that promote astrocytic secretion of pro-inflammatory cytokines and chemokines while repressing expression of anti-inflammatory cytokines (12–15). Interestingly, while both microglia and astrocytes are targets of Th1-derived soluble factors, Th17-derived soluble factors preferentially act on astrocytes (13, 15), highlighting astrocytes as central mediators of T cell-mediated neuroinflammation.

The concentration of ATP and its metabolite, adenosine, in the CNS microenvironment has emerged as a central factor in the modulation of neuroinflammation in multiple sclerosis/EAE (16). Elevated extracellular ATP (eATP) is sensed as a danger signal, promoting inflammation, while adenosine exhibits strong anti-inflammatory and immunosuppressive activities (17). The ectonucleotidases CD39 and CD73 are responsible for the conversion of ATP to adenosine. Altered expression and/or function of these enzymes have been associated to multiple sclerosis (18). Additionally, activation of the adenosine receptor A_2_AR has been shown to attenuate CNS inflammation and EAE severity (19, 20), and conversely genetic ablation of A_2_AR to exacerbate the disease (21), underscoring a key role for adenosine in controlling disease development. In support of this notion, treatment of multiple sclerosis patients or EAE mice with inosine, which similar to adenosine binds to the A_1_A, A_2_A, and A_3_A receptors, ameliorates disease onset and severity by inhibiting inflammatory cell entry into the CNS, astroglial activation and demyelination (22).

We have recently reported that deficiency of ShcC/Rai, a member of the Shc family of protein adaptors, protects mice from demyelination and prevents reactive astrogliosis during EAE notwithstanding enhanced CNS infiltration by encephalitogenic Th17 cells, due to reduced astrocytic production of pro-inflammatory molecules in response to T cell-derived factors (23). Here we have addressed the outcome of Rai deficiency on the ability of astrocytes to generate a T cell suppressive microenvironment through eATP degradation. We show that Rai-deficient astrocytes have an enhanced ectonucleotidase activity and that they upregulate CD39 expression when exposed to conditioned media from encephalitogenic T cells, which results in their enhanced ability to suppress T cells through inhibition of TCR signaling and upregulation of CTLA-4.

## Materials and Methods

### Mice

Rai^−/−^ mice in the C57BL/6J background (24, 25) and C57BL/6J controls were used. Animals were housed in a pathogen-free and climate-controlled (20 ± 2°C, relative humidity 55 ± 10%) animal facility at the University of Siena. Mice were provided with water and pelleted diet *ad libitum*. All cages are provided with environmental enrichment in the form of nesting material and mouse houses. Procedures and experimentation were carried out in accordance with the 2010/63/EU Directive and approved by the Italian Ministry of Health.

### Induction of EAE, Isolation of Glial Cells, and Generation of MOG-Specific T Cell Lines

EAE was induced in 8- to 10-week-old female mice (three Rai^−/−^ and three wild-type C57BL/6J mice) by subcutaneous injection of 200 μg MOG_35−55_ peptide emulsified in an equal volume of complete Freund's adjuvant (CFA) containing 6 mg/ml *M. tuberculosis* H37Ra (Difco Laboratories, Detroit, MI). Mice, selected by sex, age and strain, were randomly allocated to experimental groups and randomly treated. The experimental unit was single animal. We observed similar variance between the groups that were compared. On day 0 and 2 mice were injected i.p. with 300 ng *B. pertussis* toxin (Calbiochem, Darmstadt, Germany). Mice were monitored daily by two independent researchers and clinical scores were assigned according to the standard 0 to 5 scale (23, 26). Brain and spinal cords were isolated from EAE mice (15 days post-immunization) and total glial cells were obtained as described (23).

To generate MOG_35−55_ specific T cells, splenocytes and lymph nodes were harvested at day 7 after immunization with MOG_35−55_ peptide (three wild-type C57BL/6J mice) and expanded with 50 μg/ml MOG_35−55_ and 20 U/ml IL-2 in RPMI1640 with 10% BCS. After 7 days cells were re-stimulated with autologous bone marrow-derived dendritic cells, MOG_35−55_ peptide and IL-2, for 7 days. Cells underwent 2 rounds of stimulation before being used. The frequency of GM-CSF-, TNFα-, IFNγ-, or IL-17a- producing cells among MOG-T cells have been assessed by flow cytometry (% GM-CSF^+^ = 4.5 ± 1, % IL-17^+^ = 19 ± 4, % IFNγ^+^ = 55 ± 3, and % TNFα^+^ = 27 ± 0.5).

### Primary Astrocyte Culture and Treatments

Astrocyte cultures were prepared from newborn mice (15 Rai^+/+^ and 15 Rai^−/−^) as described (27). Cerebral cortices were dissociated using the Neural Tissue Dissociation kit (T) (Miltenyi Biotec, Bergisch Gladbach, Germany) and the cells were cultured in flasks. For astrocytes monoculture, supernatants containing microglia were eliminated and adherent cells were trypsinized and replated. The purity of astrocytes was ≥95% as assessed by GFAP staining.

Treatment with IFNγ (10 ng/ml) or IL-17 (50 ng/ml) was performed in serum-free medium for ATP, adenosine and phosphate measurements or in complete medium for flow cytometric analysis and qRT-PCR analysis of CD39 and CD73 expression and immunoprecipitation assays. Surface upregulation of CD39 and CD73 was analyzed in astrocytes stimulated for 120 h (peak of expression of CD39, as assessed in a preliminary time course analysis; Supplementary Figure 2) with pro-inflammatory cytokines. No surface upregulation of CD73 was found at any time point (data not shown). For the treatment with conditioned media from MOG-T cells, the culture medium was replaced with the culture supernatants from IL-2-stimulated MOG T cells in the presence or absence of a neutralizing anti-IFNγ mAb (e Bioscence). Alternatively, MOG T cells were added to astrocytes as such or previously pulsed with MOG_35−55_ peptide.

### Splenocytes, CD4^+^ T Cell Purification and Treatments

Mouse splenic mononuclear cells were separated by Mouse lympholyte gradient centrifugation (Cedarlane Laboratories, Netherlands) and resuspended in RPMI 10% BCS (two wild-type C57BL/6J mice).

Alternatively, CD4^+^ T cells were enriched from spleen using Dynabeads™ Untouched™ Mouse CD4 Cells Kit (Invitrogen).

Cells were treated with immobilized anti-CD3 (2 mg/ml; eBiosciences) and anti-CD28 (2 mg/ml; eBiosciences) mAb for 72 h, alone or in combination with either the non-hydrolyzable adenosine analog NECA (10 μM) (Sigma-Aldrich) or supernatants from IFNγ-treated Rai^−/−^ or Rai^+/+^ astrocytes, in presence or absence of the ectonucleotidase inhibitor ARL67156 (100 μM) (Sigma-Aldrich). Alternatively, cells were pre-treated with supernatants from IFNγ-treated Rai^−/−^ or Rai^+/+^ astrocytes (diluted 1:2 with culture medium) in the presence or absence of ARL67156 (100 μM) for 1 h at 37°C and activated with soluble anti-CD3 and anti-CD28 mAbs in presence or absence of 10 μM NECA.

### eATP, Adenosine, and Ectonucleotidase Activity Measurements

ATP levels in the astrocyte supernatants and cells were measured using a luciferin/luciferase assay (ATP Determination Kit A22066; Invitrogen) and a luminometer (Berthold Lumat LB 9501) according to the manufacturer's instructions. Adenosine levels were measured on astrocytes supernatants using a fluorometric assay (Adenosine Assay Kit; Cell Biolabs, INC.) and a Fluorometer (TECAN) according to the manufacturer's instructions.

For determination of nucleotide hydrolysis free phosphate was measured using the Malachite Green Phosphate Assay Kit (POMG-25H) (BioAssay Systems) at 620 nm on a microplate reader, according to the manufacturer's protocol. Specific activity was calculated using a calibration curve and expressed as nmol Pi released/mg protein/min.

Each sample was run in triplicate. Remaining cells were lysed in 0.02% SDS in phosphate-buffered saline (PBS) and protein content determined by the Pierce BCA protein assay kit (Thermo Fisher Scientific).

### Cell Lysis, Immunoprecipitations, and Immunoblots

Cells were lysed in 1% (v/v) Triton X-100 in 20 mM Tris-HCl (pH 8), 150 mM NaCl in the presence of Protease Inhibitor Cocktail Set III (Calbiochem) and 0.2 mg Na orthovanadate/ml. Postnuclear supernatants were resolved by SDS-PAGE and transferred to nitrocellulose. Alternatively, postnuclear supernatants were immunoprecipitated using RanBPM polyclonal antibody (Proteintech) and protein A Sepharose (GE Healthcare). Immunoblots were carried out using peroxidase-labeled secondary antibodies (GE Healthcare) and a chemiluminescence detection kit (Bio-rad Laboratories Inc., Milan, Italy). Immunoblots were scanned and quantitated using ImageJ software.

### Flow Cytometry and Proliferation Assays

Flow cytometric analysis of astrocytes, MOG-T cells and splenocytes was performed using AlexaFluor488-, PE-, PerCP-conjugated anti-mouse antibodies to: GFAP (clone GA5; eBioscence), CD39 (clone 24DMS1; eBioscence), CD73 (clone TY11.8; Biolegend), CTLA-4 (clone UC10-4B9; Biolegend), IL-17A (clone TC11-18H10; Becton Dickinson), IFN-γ (clone XMG1.2; Becton Dickinson), GM-CSF (clone MPI-22E9; Biolegend), TNFα (Clone MP6-XT-22; Biolegend), and isotype control antibodies. Samples were acquired on Guava Easy Cyte cytometer (Millipore) and analyzed with FlowJo software (TreeStar Inc., Ashland, OR, USA).

Proliferation was measured on CFSE loaded cells (Molecular Probes, Thermo Fisher Scientific) by flow cytometry.

### RNA Purification and RT-qPCR

Total RNA was isolated and purified from brain, astrocytes and splenocytes using the RNeasy Plus Mini Kit (Quiagen) according to the manufacturer's instructions. First-strand cDNAs were generated using the iScript™ cDNA Synthesis Kit (Bio-Rad). RT-qPCR was performed using the SsoFast™ EvaGreen® supermix kit (BIO-RAD) and specific pairs of primers listed in Supplementary Table 1.

### Statistical Analyses

One-way ANOVA with *post-hoc* Tukey or 2-way ANOVA with *post-hoc* Sidak test were used for experiments where multiple groups were compared. Mann–Whitney rank-sum tests were also performed to determine the significance of the differences between two groups. Statistical analyses were performed using GraphPad Prism Software (Version 8). A *P* < 0.05 was considered as statistically significant.

## Results

### Rai Dampens CD39 Enzyme Activity in Astrocytes in Response to IFNγ Treatment

**Figure 1:**
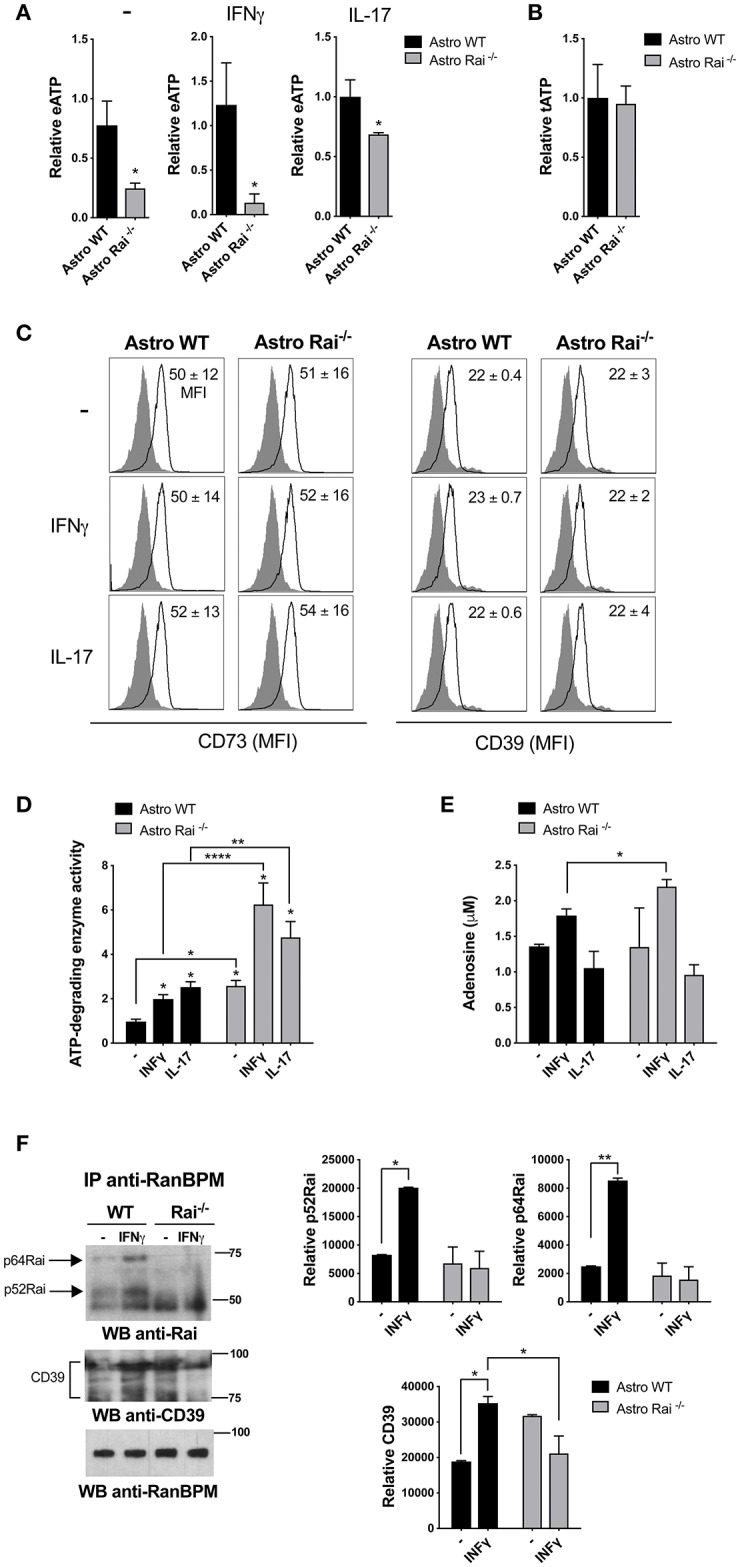
Rai dampens extracellular ATP-degrading enzyme activity in astrocytes. **(A)** ATP (eATP) quantification in culture supernatants from Rai^+/+^ (Astro WT) and Rai^−/−^ (Astro Rai^−/−^) astrocytes stimulated for 5 h with IFNγ (10 ng/ml) or IL-17 (50 ng/ml) or left untreated (-). Data are presented as mean ± SD of relative luciferase units (RLU) in supernatants from Rai^−/−^ astrocytes vs. Rai^+/+^ astrocytes. Data have been normalized to the mean RLU value of Rai^+/+^ astrocytes (*n* = 5). **(B)** Total ATP (tATP) content in unstimulated Rai^+/+^ (Astro WT) and Rai^−/−^ (Astro Rai^−/−^) astrocytes. **(C)** Flow cytometric analysis of surface CD73 and CD39 in Rai^+/+^ (Astro WT) and Rai^−/−^ (Astro Rai^−/−^) astrocytes stimulated for 5 h with IFNγ (10 ng/ml), IL-17 (50 ng/ml) or left untreated (-). Data are presented as mean ± SD of mean fluorescence intensity (MFI) (*n* = 4). **(D)** Quantification of enzymatic activities of extracellular ATP-degrading enzymes in Rai^+/+^ (Astro WT) and Rai^−/−^ (Astro Rai^−/−^) astrocytes stimulated with IL-17 or IFNγ for 5 h or left untreated (-), then depleted of their culture supernatant and incubated with 1 mM ATP. Data are presented as mean fold change ± SD of specific enzymatic activities (nmol free phosphate/mg protein/min) in Rai^+/+^ astrocytes and Rai^−/−^ astrocytes, with unstimulated Rai^+/+^ astrocytes taken as 1 (*n* = 3). **(E)** Quantification of adenosine in culture supernatants of astrocytes treated as in D. Data are presented as mean ± SD of adenosine concentration (μM) (*n* = 3). **(F)** Immunoblot analysis with anti-Rai or anti-CD39 antibodies of RanBPM-specific immunoprecipitates from total cell lysates of Rai^+/+^ and Rai^−/−^ astrocytes treated with IFNγ (10 ng/ml) for 15 min (*n* = 2). The quantification by laser densitometry of the levels of each of the proteins normalized to the level of RanBPM in each sample is shown (*n* = 2). 2-Way ANOVA and Mann–Whitney test ^****^p < 0.0001, ^**^p < 0.01, ^*^p < 0.05.

To address the impact of Rai deficiency on the eATP-degrading activity of astrocytes, ATP was quantified in culture supernatants from Rai^+/+^ and Rai^−/−^ astrocytes generated from newborn mice brain, stimulated or not with IL-17 or IFNγ. Lower levels of eATP were found in culture supernatants of Rai^−/−^ astrocytes compared to Rai^+/+^ astrocytes, despite the fact that the total levels of ATP were comparable (Figures 1A,B). No differences in surface CD39/CD73 expression were observed under these conditions (Figure 1C, Supplementary Figure 1), suggesting that Rai might modulate the eATP-degrading activity of astrocytes.

To test this possibility we compared the ATP-degrading activity of control and Rai^−/−^ astrocytes stimulated or not with IL-17 or IFNγ by incubating cells depleted of their culture supernatant with 1 mM ATP and measuring free phosphate production. Both cytokines promoted ATP-degradation, with Rai^−/−^ astrocytes hydrolyzing ATP more efficiently, both under basal conditions and following IFNγ or IL-17 treatment (Figure 1D). Quantification of adenosine in culture supernatants from astrocytes added with exogenous ATP showed that IFNγ, but not IL-17, enhanced adenosine production, which was further enhanced by Rai deficiency (Figure 1E). These data indicate that IFNγ modulates adenosine generation by astrocytes and that Rai dampens the activity of ATP-degrading enzymes in these cells.

To translate these results to the context of EAE we measured the ATP-degrading activity of astrocytes isolated from the spinal cord of Rai^−/−^ and control EAE mice. Similar to the results obtained on astrocytes derived from the brain of newborn mice, Rai^−/−^ astrocytes obtained from the CNS of EAE mice degraded ATP more efficiently compared to their wild-type counterparts (Supplementary Figure 3). These data suggest that the protective role of Rai deficiency in astrocytes toward neuroinflammation in EAE could be dependent at least in part on the ability of Rai to negatively control eATP degradation and adenosine generation.

### Rai Couples CD39 to its Negative Regulator RanBPM

To address the mechanism responsible for the ability of Rai to negatively control eATP degradation we focused on the rate-limiting enzyme of the cascade which converts ATP/ADP to adenosine, namely CD39. Since the scaffolding protein RanBPM binds to the cytosolic tail of CD39 and downregulates its ectonucleotidase activity (28), we hypothesized that Rai may participate in this molecular complex to restrain CD39 function. Rai^+/+^ and Rai^−/−^ astrocytes were left untreated or were treated with IFNγ and post-nuclear supernatants were immunoprecipitated with anti-RanBPM antibodies. RanBPM-specific immunoprecipitates were analyzed by immunoblotting with anti-Rai and anti-CD39 Abs. Rai was found to associate with RanBPM in response to IFNγ (Figure 1F). Interestingly, the IFNγ-dependent association of RanBPM with CD39 was reduced in Rai^−/−^ astrocytes compared to control astrocytes (Figure 1F), indicating that IFNγR signaling promotes CD39 activation and suggesting that Rai limits CD39 activity by promoting RanBPM recruitment to CD39.

### Rai Negatively Controls the Contact-Dependent and -Independent Upregulation of CD39 and CD73 Elicited by Encephalitogenic T Cells

**Figure 2:**
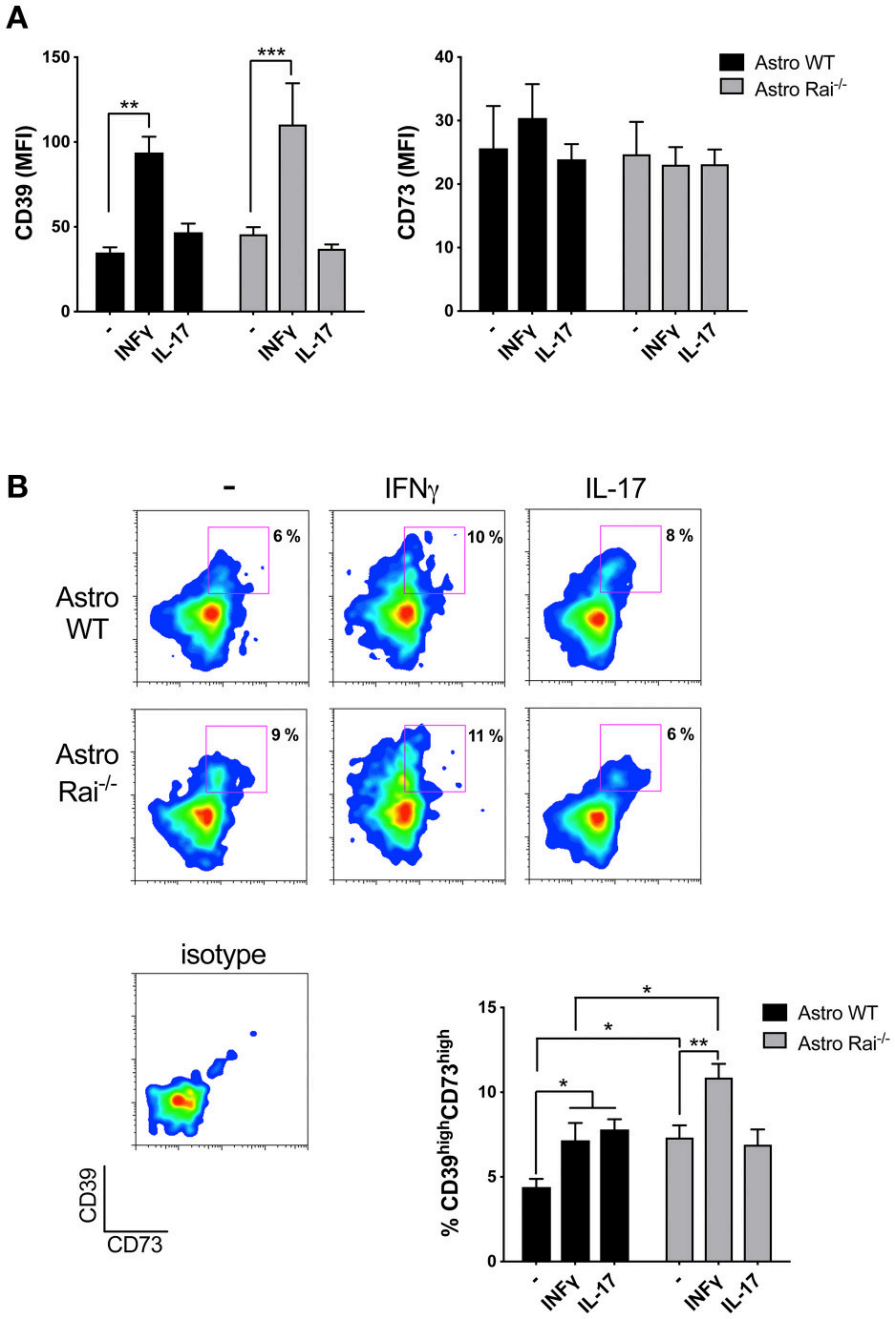
Rai limits the IFNγ-dependent upregulation of CD39 on astrocytes. Flow cytometric analysis of surface CD73 and CD39 in Rai^+/+^ (Astro WT) and Rai^−/−^ (Astro Rai^−/−^) astrocytes untreated (-) or stimulated for 120 h with IFNγ (10 ng/ml) or IL-17 (50 ng/ml). Data are presented as mean ± SD of mean fluorescence intensity (MFI) **(A)** or frequency of CD39^high^CD73^high^ astrocytes **(B)**. Representative dot plots of CD39 and CD73 are shown (*n* > 3). Two-Way ANOVA, ^***^p < 0.001, ^**^p < 0.01, ^*^p < 0.05.

Astrocytes have been demonstrated to suppress recently activated CD4^+^ T cells by inducing the upregulation of CD39/CD73 on their surface, which correlates with the acquisition of an immunosuppressive Th17 phenotype (8). Whether inflammatory T cells can in turn affect the expression of these ATP-degrading enzymes in astrocytes, and the role of Rai in this process, have as yet not been explored. At present no published data are available on surface expression of CD39 and CD73 in mouse primary astrocytes either in the basal state or in response to cytokines. Surface CD39 and CD73 was measured by flow cytometric analysis of control and Rai^−/−^ astrocytes following prolonged (120 h) treatment with IL-17 or IFNγ. IL-17 had no effect on either CD39 or CD73 surface expression (Figure 2A). At variance, IFNγ was found to promote CD39 upregulation with, a slight, yet not significant, further increase in Rai^−/−^ astrocytes compared to control astrocytes (Figure 2A). The increase in surface CD39 correlated with an increase in the levels of CD39 mRNA, as assessed by RT-qPCR. An increase in the levels of CD73 mRNA was also observed in Rai^−/−^ astrocytes compared to control astrocytes, however this was not paralleled by an global concomitant increase in surface CD73 (Supplementary Figure 4). Interestingly, co-upregulation of surface CD39 and CD73 was observed in response to both IFNγ and IL-17 in a small subpopulation of astrocytes (Figure 2B). This CD39^high^CD73^high^ subpopulation was larger in Rai^−/−^ astrocytes compared to wild-type controls under steady-state conditions and was further expanded following IFNγ, but not IL-17, treatment (Figure 2B).

**Figure 3:**
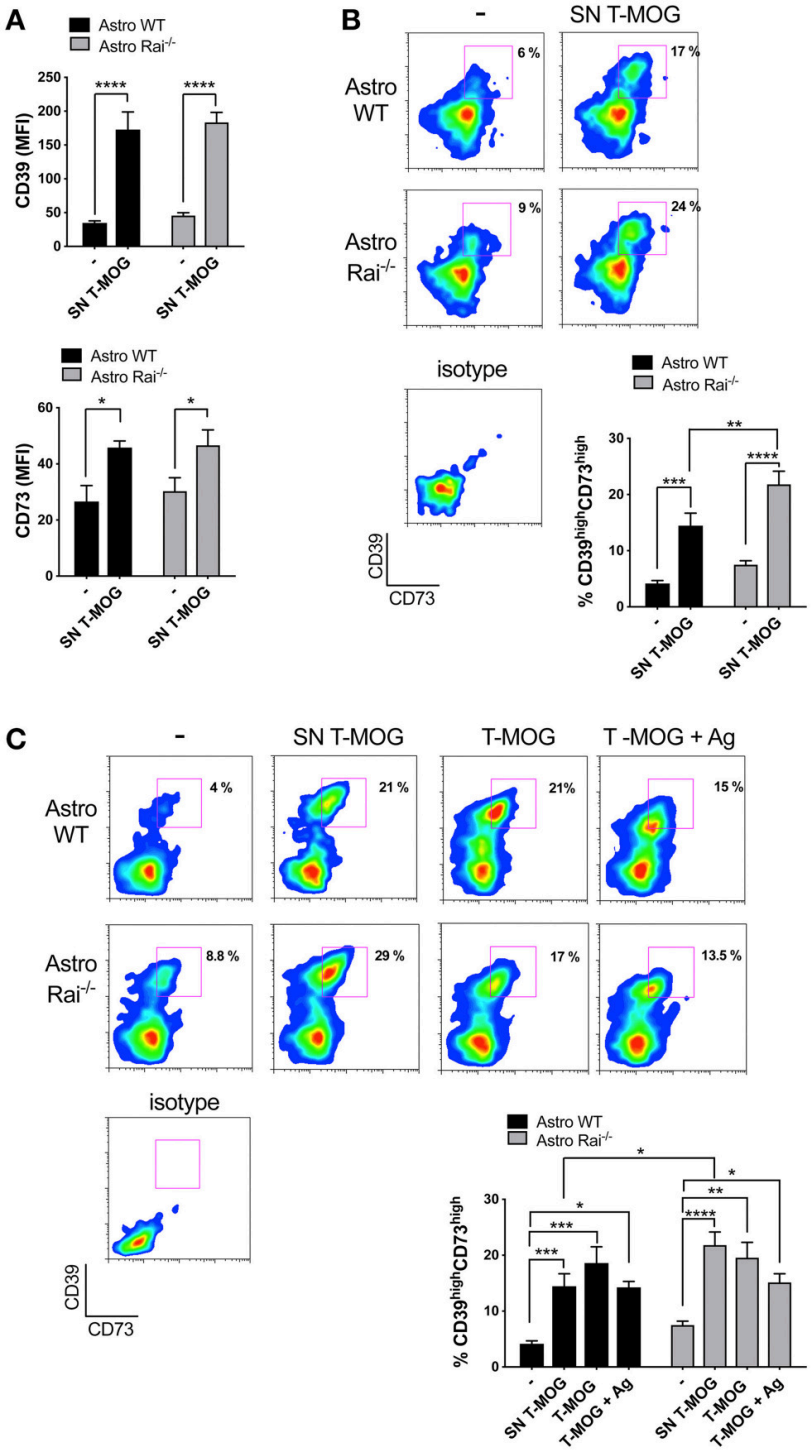
Rai negatively controls the contact-dependent and -independent upregulation of CD39 and CD73 elicited by encephalitogenic T cells. **(A,B)** Flow cytometric analysis of surface CD73 and CD39 in Rai^+/+^ (Astro WT) and Rai^−/−^ (Astro Rai^−/−^) astrocytes untreated (-) or stimulated for 120 h with culture supernatants from MOG-specific T cells generated from WT mice (SN T-MOG). Data are presented as mean ± SD of mean fluorescence intensity (MFI) **(A)** or frequency of CD39^high^CD73^high^ astrocytes (*n* > 3) **(B)**. **(C)** Flow cytometric analysis of surface CD73 and CD39 expression in Rai^+/+^ (Astro WT) and Rai^−/−^ (Astro Rai^−/−^) astrocytes treated for 120 h with either culture supernatants from MOG-specific T cells generated from WT mice (SN T-MOG) or MOG-specific T cells depleted of their culture supernatants (T-MOG) in the presence (+Ag) or absence of MOG antigen. Representative dot plots are shown. The histograms show the frequency of the CD39^high^CD73^high^ population. Data are presented as mean ± SD of the percentage of CD39^high^CD73^high^ cells (*n* > 3). Representative dot plots are shown. Two-Way ANOVA, ^****^p < 0.0001, ^***^p < 0.001, ^**^p < 0.01, ^*^p < 0.05.

To mimic the CNS microenvironment shaped by infiltrating T cells during EAE, surface CD73 and CD39 were measured by flow cytometry on wild-type and Rai^−/−^ astrocytes treated with conditioned media from MOG-specific T cells for 120 h. Under these conditions both CD39 and, to a lesser extent, CD73, were upregulated in both wild-type and Rai^−/−^ astrocytes (Figure 3A). Additionally, a substantial increase in the abundance of the CD39^high^CD73^high^ wild-type astrocyte subpopulation was observed, with a further significant increase in Rai^−/−^ astrocytes (Figure 3B). No significant effect on the frequency of CD39^high^CD73^high^ astrocytes was observed when an anti-IFNγ antibody was added to the conditioned media (data not shown), suggesting that other T cell-derived factors are responsible for the robust co-upregulation of CD39 and CD73 on this astrocyte subpopulation. These results indicate that encephalitogenic T cells promote co-upregulation of CD39 and CD73 on astrocytes in a contact-independent manner and that Rai deficiency results in an enhanced ability of astrocytes to respond to T cell-derived factors.

To understand whether surface ectonucleotidase expression on astrocytes can be further modulated by their physical contact with T cells, surface CD39 and CD73 were measured on wild-type and Rai^−/−^ astrocytes co-cultured for 120 h in the presence or absence of MOG with encephalitogenic T cells previously depleted of their culture supernatant. Under these conditions an increase in the abundance of the CD39^high^CD73^high^ astrocyte subpopulation was observed, independently of the presence of antigen (Figure 3C). However, Rai deficiency did not affect the contact-dependent upregulation of CD39 or CD73, as opposed to the enhancement observed in the presence of conditioned media from MOG-specific T cells (Figure 3C). Hence, encephalitogenic T cells elicit a co-upregulation of CD39 and CD73 on astrocytes in both a contact-independent and a contact-dependent but antigen-independent manner, and the contact-independent response is enhanced in astrocytes lacking Rai.

### Rai^−/−^ Astrocytes Inhibit T Cell Proliferation by Suppressing TCR Signaling and Promoting Adenosine-Dependent CTLA-4 Upregulation

**Figure 4:**
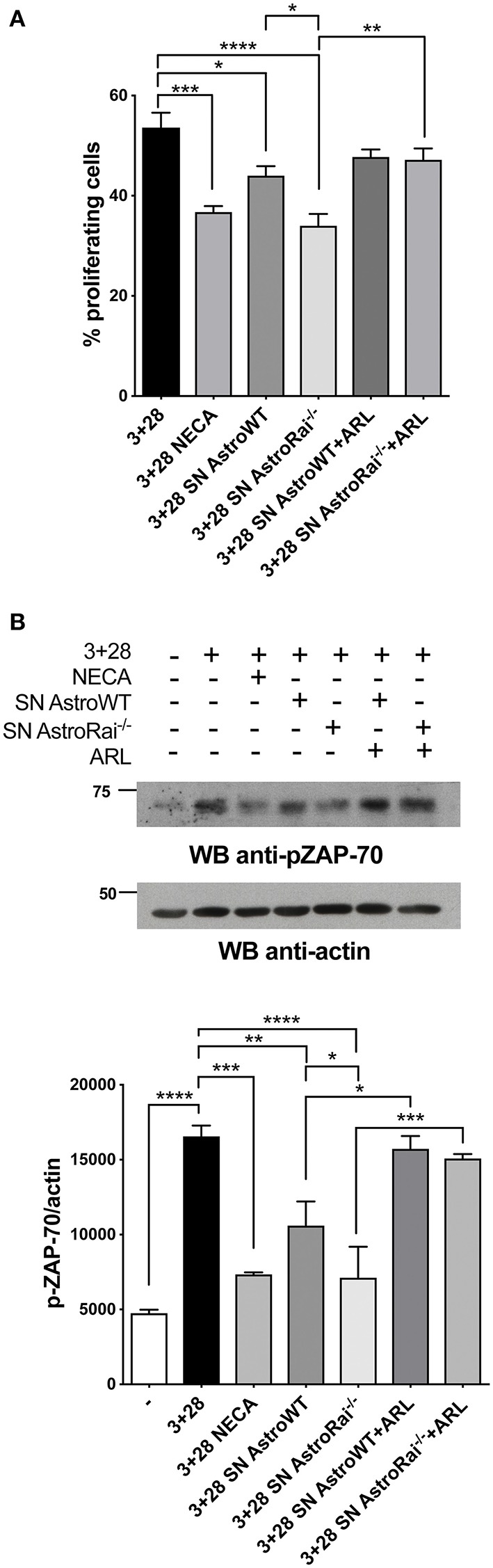
Rai^−/−^ astrocytes inhibit T-cell proliferation and TCR signaling through cell-cell contact-independent mechanisms. **(A)** Flow cytometric analysis of CFSE-labeled splenic mouse cells from wild-type mice stimulated for 72 h with anti-CD3/CD28 antibodies (3+28) in combination with either NECA or supernatants from IFNγ-treated Rai^−/−^ (SN AstroRai^−/−^) or Rai^+/+^ (SN AstroWT) astrocytes in presence or absence of ARL67156 (100 μM) (ARL). The graph shows the mean value ± SD of the percentage of CFSE^low^ cells (proliferating cells) (*n* = 5). **(B)** Immunoblot analysis of ZAP-70 phosphorylation in postnuclear supernatants of splenocytes from wild-type mice stimulated for 5 min with anti-CD3/CD28 antibodies (3+28) in combination with either NECA or supernatants from IFNγ-treated Rai^−/−^ (SN AstroRai^−/−^) or Rai^+/+^ (SN AstroWT) astrocytes in presence or absence of ARL67156 (100 μM) (ARL). A control blot of the same filter is shown. The histogram shows the quantification by densitometric analysis of the levels of phosphorylated ZAP-70 relative to actin (*n* = 3). One-way ANOVA; ^****^p < 0.0001, ^***^p < 0.001, ^**^p < 0.01, ^*^p < 0.05.

The enhanced ability of Rai^−/−^ astrocytes to co-upregulate surface CD39/CD73 and hydrolyze eATP in the presence of the pro-inflammatory cytokines or factors released by encephalitogenic T cells suggests that Rai^−/−^ astrocytes may suppress the activity of infiltrated T cells in an adenosine-dependent manner. To test this hypothesis we measured the proliferation of splenic T cells activated by CD3/CD28 costimulation in the presence of conditioned media from IFNγ-treated Rai^−/−^ or Rai^+/+^ astrocytes, using a non-hydrolysable adenosine analog as control. Flow cytometric analysis of CFSE-labeled splenocytes showed that culture supernatants from both IFNγ-treated wild-type and Rai^−/−^ astrocytes, but not from untreated astrocytes, inhibited T cell proliferation (Figure 4A, Supplementary Figure 5). These effects were neutralized by treatment with the CD39/CD73 inhibitor ARL67156 (Figure 4A), indicating that they were mediated by adenosine. ARL67156 alone had no effect on CD3/CD28-dependent proliferation (Supplementary Figure 5). Suppression of T cell proliferation was more profound in the presence of conditioned media from Rai^−/−^ astrocytes (Figure 4A), consistent with their higher adenosine content (Figure 1).

Adenosine binding to A_2_AR results in an elevation in intracellular cAMP, which effectively inhibits TCR signaling through the PKA-dependent activation of the kinase Csk, a negative regulator of the initiating kinase Lck (29). Lck is required for the phosphorylation-dependent recruitment of the kinase ZAP-70 to the TCR, a key step for signal propagation (30). To explore the ability of astrocytes to modulate TCR signaling through their ATP-hydrolysing activity, splenic T cells were activated by CD3/CD28 costimulation in the presence of conditioned media from wild-type or Rai^−/−^ astrocytes in the presence or absence of ARL67156, and the activation of ZAP-70 was measured by immunoblot using a phosphospecific antibody. Consistent with their inhibitory effect on T cell proliferation, supernatants from IFNγ-treated, but not from untreated, astrocytes suppressed ZAP-70 activation, with a higher efficiency for supernatants from Rai^−/−^ astrocytes (Figure 4B, Supplementary Figure 5). Inhibition was fully relieved by the ectonucleotidase inhibitor (Figure 4B), supporting the notion that suppression of TCR signaling by astrocyte-derived factors is mediated by CD39/CD73-dependent adenosine production.

**Figure 5:**
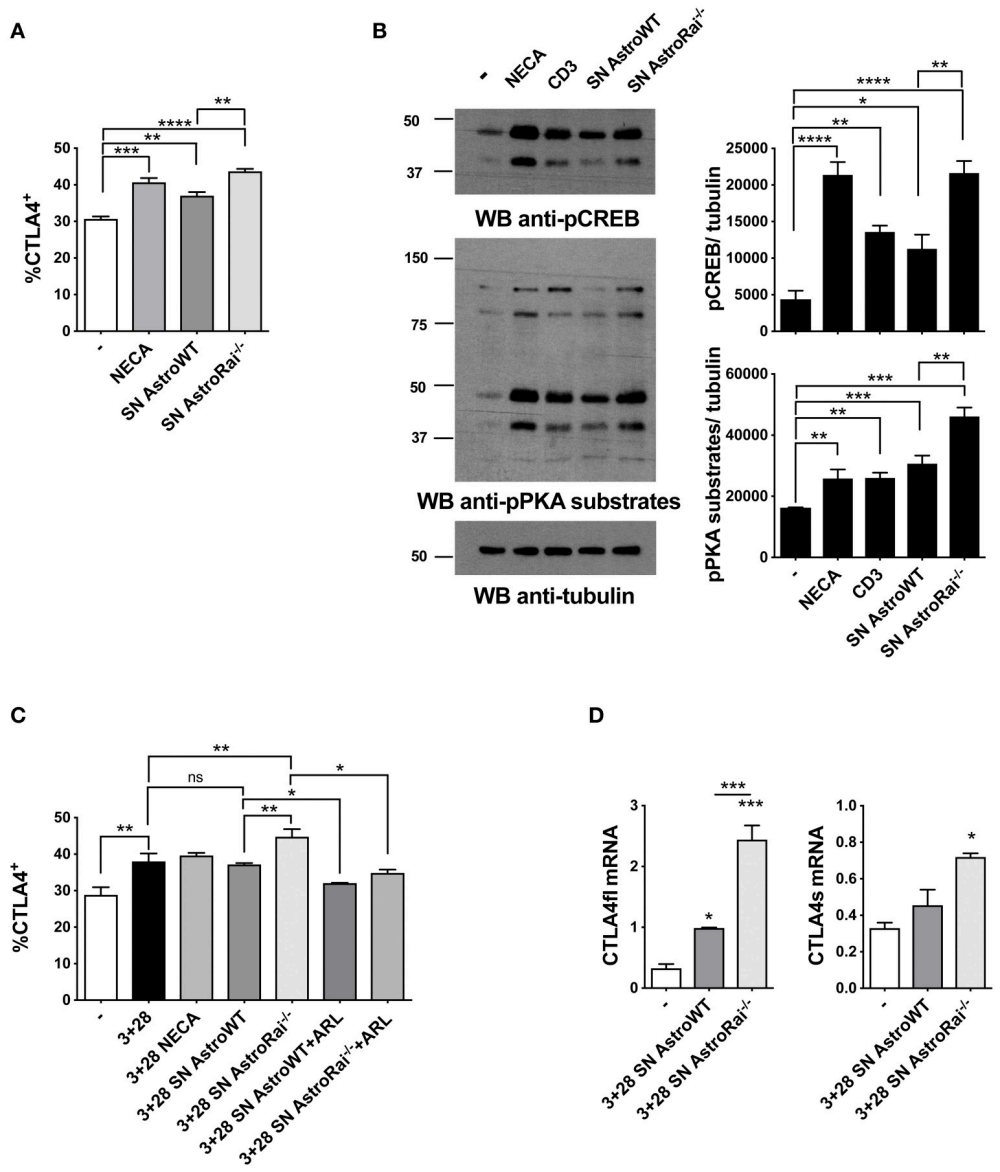
Rai^−/−^ astrocytes promote CTLA-4 expression on T cells through cell-cell contact-independent mechanisms. **(A)** Flow cytometric analysis of the frequency of CTLA-4 positive cells among splenic mouse T cells treated with culture supernatants from IFNγ-treated Rai^+/+^ (SN AstroWT) or Rai^−/−^ (SN AstroRai^−/−^) astrocytes or NECA for 72 h. Data are presented as mean value ± SD of the percentage of CTLA-4 positive cells (*n* = 3). **(B)** Immunoblot analysis of phosphorylated PKA substrates and phospho-CREB in lysates of CD4^+^ T cells from wild-type mice stimulated with anti-CD3 antibodies, culture supernatants from IFNγ-treated Rai^+/+^ (SN AstroWT) or Rai^−/−^ (SN AstroRai^−/−^) astrocytes or NECA for 5 min. β-Tubulin was used as loading control. The histogram shows the quantification by densitometric analysis of the levels of phosphorylated PKA substrates and CREB relative to tubulin (*n* = 3). **(C)** Flow cytometric analysis of the frequency of CTLA-4 positive cells among splenic mouse T cells stimulated with anti-CD3/CD28 antibodies (3+28) in combination with either NECA or supernatants from IFNγ-treated Rai^+/+^ (SN AstroWT) or Rai^−/−^ (SN AstroRai^−/−^) astrocytes in presence or absence of ARL67156 (100 μM) (ARL). Data are presented as mean value ± SD of the percentage of CTLA-4 positive cells (*n* = 5). **(D)** Real-Time PCR analysis of full length and soluble CTLA-4 mRNA expression in splenic mouse cells stimulated for 24 h with anti-CD3/CD28 antibodies (3+28) in the presence of supernatants from IFNγ-treated Rai^+/+^ (SN AstroWT) or Rai^−/−^ (SN AstroRai^−/−^) astrocytes. The levels of the different transcripts were normalized to GAPDH, used as housekeeping gene. Data are presented as mean value ± SD (*n* = 3). One-way ANOVA; ^****^p < 0.0001, ^***^p < 0.001, ^**^p < 0.01, ^*^p < 0.05.

In addition to directly inhibiting TCR signaling, adenosine suppresses T cell responses by inducing the cAMP/PKA-dependent expression of the inhibitory receptor CTLA-4, which blocks CD28-mediated costimulation (31, 32). Additionally, an upregulation of CTLA-4 expression has been reported in T cells exposed to astrocytes or to their conditioned media (7), suggesting a mechanistic link between these observations and our finding that astrocytes effectively degrade eATP. To address this issue, surface CTLA-4 was measured by flow cytometry on T cells exposed to conditioned media from IFNγ-treated wild-type or Rai^−/−^ astrocytes. The levels of T cell surface CTLA-4 were higher in the presence of IFNγ-treated astrocyte culture supernatants, similar to adenosine-treated T cells. Significantly higher levels of CTLA-4 were observed in the presence of supernatants from Rai^−/−^ astrocytes compared to wild-type astrocytes (Figure 5A). The enhanced ability of conditioned media from IFNγ-treated Rai^−/−^ astrocytes to induce A_2_AR signaling compared with wild-type astrocytes was further supported by an enhancement of PKA activity and CREB phosphorylation (Figure 5B). Culture supernatants from untreated astrocytes had no effect on surface CTLA4 (Supplementary Figure 5). Supernatants from IFNγ-treated Rai^−/−^ astrocytes, but not from IFNγ-treated WT astrocytes, induce significant CTLA4 upregulation also on CD3/CD28 activated T cells when comparing with activated T cells without addition of astrocytes supernatant (Figure 5C). This effect was completely neutralized by the ectonucleotidase inhibitor (Figure 5C), indicating that CTLA-4 upregulation by astrocytes is dependent on their ectonucleotidase activity. ARL67156 alone had no effect on CD3/CD28-dependent CTLA4 upregulation (Supplementary Figure 5).

While CTLA-4 can be rapidly expressed at the T-cell surface through the release of an intracellular pool stored in lysosomes (33), adenosine-dependent CTLA-4 upregulation involves *de novo* gene expression triggered by cAMP-dependent activation of the transcription factor CREB (34). To understand whether the increase in surface CTLA-4 observed in the presence of astrocyte culture supernatants was the result of transcriptional activation, we measured CTLA-4 mRNA levels on splenic T cells activated by CD3/CD28 costimulation in the presence of conditioned media from IFNγ-treated wild-type or Rai^−/−^ astrocytes. Real-time RT-PCR analysis revealed an increase in the levels of the transcript for both the full length and the soluble form of CTLA-4, which results from alternative splicing and inhibits T-cell responses by binding B7 on APCs (35, 36) (Figure 5D). Collectively, these results indicate that astrocytes inhibit T cell activation and proliferation by suppressing TCR signaling and enhancing CTLA-4 expression through CD39/CD73-mediated adenosine production and cAMP/PKA signaling, which are enhanced in the absence of Rai.

### A2 Reactive Astrocytes Are Induced by Encephalitogenic T Cells in the Absence of Rai

Astrocytes can polarize toward a neurotoxic A1 phenotype or a neuroprotective A2 anti-inflammatory phenotype depending on the disease. To date the signaling mechanisms responsible for the shift elicited by inflammatory cues remains largely unknown (11), and whether encephalitogenic T cells drive astrocyte polarization has as yet not been explored.

**Figure 6:**
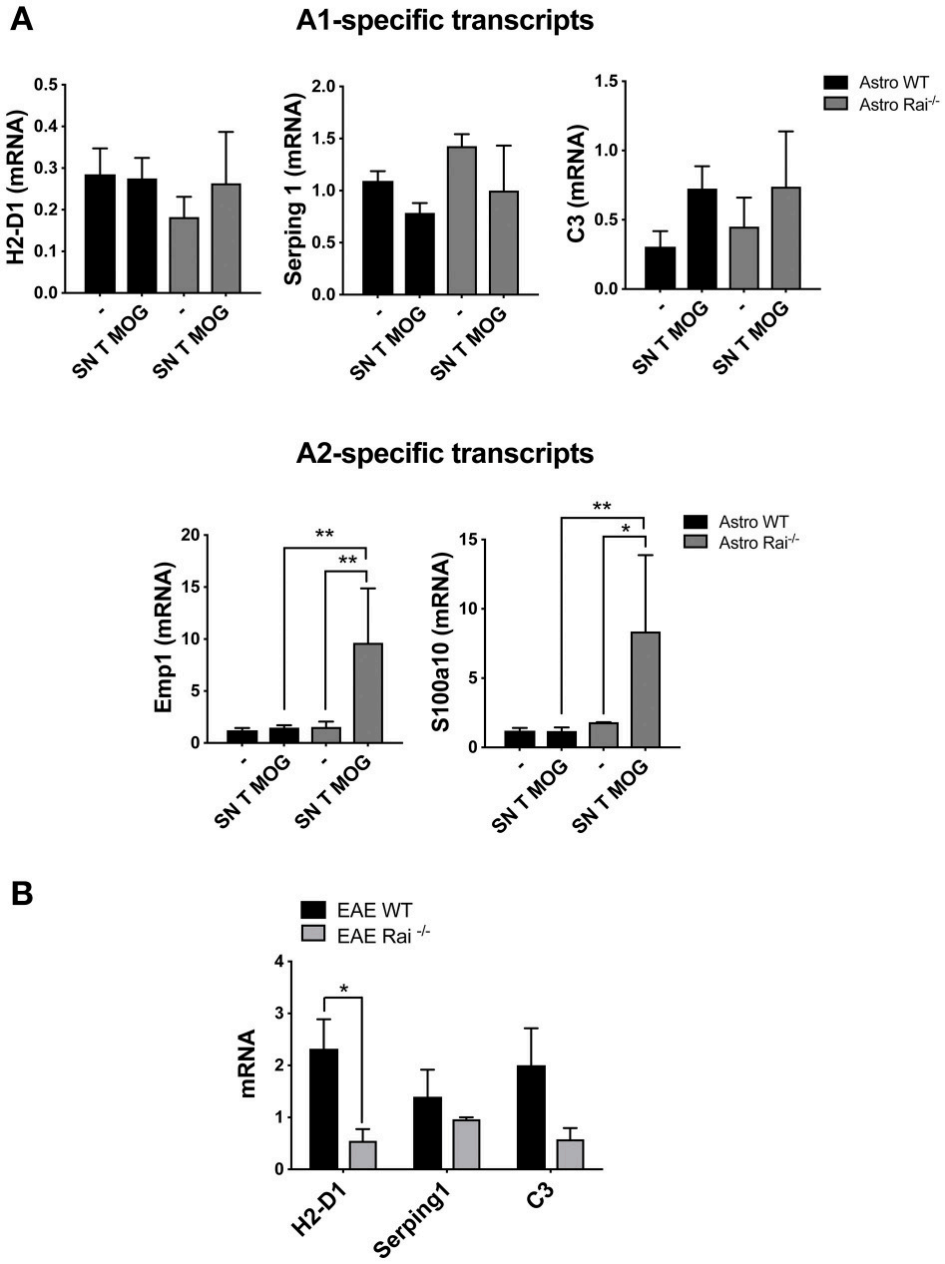
Rai negatively controls the conversion of astrocytes toward a neuroprotective phenotype. **(A)** Real-Time PCR analysis of A1-specific (H2-D1, Serping and C3; upper panels) or A2-specific (Emp1 and S100a10; lower panels) transcripts in WT (Astro WT) and Rai^−/−^ (Astro Rai^−/−^) astrocytes either untreated or treated for 24 h with culture supernatants from MOG-specific T cells generated from WT mice. Data from 3 independent experiments, each carried out on the pooled astrocytes from at least 5 Rai^+/+^ or 5 Rai^−/−^ mice, are presented as mean value ± SD. The levels of the different transcripts were normalized to GAPDH, used as housekeeping gene. **(B)** Real-Time PCR analysis of the A1-specific transcripts H2-D1, Serping and C3 in the brain of Rai^+/+^ EAE mice (EAE WT) and Rai^−/−^ EAE mice (EAE Rai^−/−^) 15 days post immunization. Data are presented as mean value ± SD obtained on three Rai^+/+^ and three Rai^−/−^ EAE mice. 2-way ANOVA; ^**^p < 0.01, ^*^p < 0.05.

To investigate whether soluble factors released by encephalitogenic T cells induce astrocyte polarization, A1 or A2-specific transcripts were measured in astrocytes cultured for 24 h in conditioned media from encephalitogenic T cells. While no effect was detected in control astrocytes, under these conditions a strong induction of the A2-specific transcripts Emp1 and S100a10 was detected in Rai^−/−^ astrocytes. At variance, Rai deficiency did not affect the levels of the A1-specific transcript H2-D1 and Serping1 (Figure 6A). Consistent with the protective role played by Rai deficiency in the EAE mouse model, lower levels of the A1-specific transcript H2-D1, Serping1 and C3 were found in the brain of Rai^−/−^ EAE mice compared with control EAE mice (Figure 6B). These data identify Rai as a signaling molecule that restrains the polarization of astrocytes to the neuroprotective A2 phenotype.

## Discussion

The cross-talk of astrocytes with encephalitogenic T cells is centrally implicated in multiple sclerosis pathogenesis (13, 37). Astrocytes respond to Th1 and Th17 cell-derived cytokines by producing factors that attract inflammatory cells. Additionally, they act as APC to promote effector T cell activation and expansion (38). However, activated astrocytes also deploy a variety of strategies to counteract inflammation and limit neuronal damage, including induction of Fas-mediated apoptosis of infiltrated T cells, skewing of T-cell polarization to a protective Th2 phenotype and Treg-dependent suppression of encephalitogenic T-cells (10, 39, 40). Here we document a new protective mechanism exploited by astrocytes to suppress T-cell activation and proliferation, which involves the upregulation of the astrocytic expression and activity of the ectonucleotidases CD39 and CD73 in response to pro-inflammatory factors released by encephalitogenic T cells. Additionally, we identify Rai as a negative regulator of this inhibitory circuitry.

The balance between eATP and adenosine has emerged as an important factor in the control of neuroinflammation to which both infiltrating T cells and astrocytes contribute. eATP boosts T-cell activation and promotes Th17 cell differentiation while inhibiting Treg cell differentiation and stability (41). Additionally eATP triggers microglia activation (42). On the other hand, ATP degradation to adenosine is a potent mechanism of T-cell suppression, and in fact CD39 has been established as a Treg cell marker that contributes to their inhibitory function (18, 43, 44). The function of CD39^+^ Treg cells in MS is still unclear. Indeed in relapsing-remitting multiple sclerosis enhanced frequency of CD39^+^ Treg cells has been reported both during relapse (45, 46) and during the remission phase (47). Adenosine suppresses TCR signaling by interacting with the adenosine receptor A_2_AR (48), which activates a cAMP/PKA axis that inhibits TCR signaling at multiple steps (29). We found that conditioned media from IFNγ-activated astrocytes were able to inhibit T-cell proliferation and that this effect was abrogated by an ectonucleotidase inhibitor, indicating a contact-independent, adenosine-mediated mechanism of T-cell suppression. Accordingly, proximal TCR signaling, which requires activation of the kinase Lck that is inhibited by cAMP (49), was impaired when T cells were activated in the presence of conditioned media from astrocytes. The ability of the ectonucleotidase inhibitor to reverse this effect highlights a major role for the ATP-degrading, adenosine-elevating activity of CD39 in T-cell suppression by astrocytes.

Interestingly, we found that the ATP-degrading activity of astrocytes contributes to the suppression of T-cell proliferation through an additional, cAMP-dependent mechanism involving upregulation of the inhibitory receptor CTLA-4. Astrocytes have been shown to induce the contact-independent CTLA-4 upregulation on activated T cells, suggesting the presence of soluble inhibitory factors (7). The fact that the enhancing effect of culture supernatants from IFNγ-activated astrocytes on T cell expression of CTLA-4 can be reversed by an ectonucleotidase inhibitor supports the notion that a major one among these factors is adenosine. Of note, we found that surface CTLA-4 upregulation was paralleled by an increase in the levels of specific transcripts, consistent with the fact that A_2_AR triggering on T cells promotes CTLA-4 transcription through its cAMP-elevating activity and the resulting activation of the transcription factor CREB (34). Indeed, we found that conditioned media from IFNγ-treated astrocytes were able to trigger CREB activation in an ectonucleotidase-dependent manner.

Interestingly, the levels of surface CD39 expression were upregulated in response to long-term treatment with IFNγ but not IL-17, while surface CD73 expression was not affected, highlighting CD39 as a limiting factor in ATP degradation by astrocytes and indicating that this protective response may be elicited preferentially by Th1 cells. It is however noteworthy that both IFNγ and IL-17, and to an even greater extent conditioned media from encephalitogenic T cells, increase the abundance of a CD39^+^ astrocyte subpopulation that co-expresses CD73, which may account for the increased ATP-degrading activity detected under these conditions. This finding supports the notion that astrocytes shift toward an immunosuppressive phenotype in a Th1/Th17-conditioned microenvironment. Of note, transcription of the gene encoding CD39 has been reported to be activated by cAMP (50). Taking into account the fact that astrocytes are able to promote CD39 upregulation on co-cultured activated T cells (8), a possible scenario is that the resulting adenosine-generating activity of T cells may trigger adenosine signaling on astrocytes, thereby promoting cAMP accumulation and transcriptional activation of CD39, which would in turn result in suppressive adenosine-mediated signaling in T cells. ROS-dependent upregulation of CD39 has been recently reported in CD8 T cells (51). While the impact of ShcC/Rai on ROS production has as yet not been investigated, our finding that surface CD39 expression was upregulated in response to long-term treatment with IFNγ opens the possibility that enhanced ROS generation may account for the higher CD39 expression also in astrocytes.

Our results identify Rai as a negative regulator of astrocyte-mediated, adenosine-dependent T-cell suppression. Indeed, the T cell suppressive effects of conditioned media from IFNγ-treated astrocytes were enhanced by Rai deficiency. This results both from the enhanced ability of Rai^−/−^ astrocytes to degrade eATP to adenosine in response to short-term IFNγ treatment and from the greater increase in CD39 expression and frequency of the CD39^+^CD73^+^ subpopulation after long-term IFNγ treatment compared to their wild-type counterparts. These results provide insights into the mechanisms responsible for the protective effect of Rai deficiency in astrocytes from encephalitogenic T cell-dependent neurodegeneration (23). Rai was initially identified as a molecular adaptor that couples the receptor tyrosine kinase Ret to Akt in neuronal cells (52). We showed that in T cells Rai limits antigen receptor signaling by impairing ZAP-70 recruitment to the activated TCR (53). The restraining effects of Rai on IFNγ-dependent CD39 expression and activity could be hypothesized to result from a similar mechanism involving the ability of Rai to exploit its adaptor function to interfere with IFNγR signaling.

That Rai is able to modulate the activity CD39 is intriguing. Studies on this ectoenzyme have been largely focused on the extracellular domain, which represents the most conspicuous part of the protein (54). Interestingly, recent evidence indicates that the short cytosolic tail is also implicated in the regulation of CD39 activity. Namely, RanBPM, an interactor of the small GTPase Ran, has been shown to associate with the cytosolic tail of CD39, which negatively regulates its activity, in B cells (28). RanBPM acts as a scaffolding protein, interacting with a variety of membrane proteins and receptors (55). Here we demonstrate that Rai forms a complex with RanBPM and promotes IFNγ-dependent recruitment of RanBMP to CD39, thereby restraining its function, which places Rai in the negative regulatory circuitry of CD39, accounting for the enhanced CD39 activity in Rai^−/−^ astrocytes.

Reactive astrocytes may adopt two distinct phenotypes, A1 and A2, with A1 astrocytes being neurotoxic and A2 astrocytes neuroprotective (11). Although astrocyte conversion to the A1 phenotype has been shown to be modulated by activated microglia in human neurodegenerative diseases including multiple sclerosis (11), the underlying mechanism and the impact of encephalitogenic T cells on this process remain unknown. Our data provide evidence for a new role of Rai as a negative regulator of astrocyte polarization to the A2 phenotype, highlighting an additional mechanism involving astrocytes that contributes to attenuating EAE severity in Rai^−/−^ mice (23).

In conclusion, the results presented in this report show a reciprocal interplay whereby pathogenic T cells trigger CD39 expression and activity on astrocytes, highlighting this ectonucleotidase as a hub where signals from T cells and astrocytes converge to modulate the pathogenic activity of T cells in the CNS. They moreover identify astrocytic Rai as a central player in this cross-talk which unleashes the pathogenic effects of infiltrated encephalitogenic T cells in the CNS by negatively regulating a protective CD39-based T cell suppression circuitry. Finally, they provide evidence that Rai negatively regulates the polarization of reactive astrocytes toward a neuroprotective A2 phenotype. Both the enhanced T cell suppressive activity of Rai-deficient astrocytes and their enhanced A2 polarization are likely to account for our finding that Rai deficiency in astrocytes prevents reactive astrogliosis and ameliorates EAE (23).

## Ethics Statement

This study was carried out in accordance with the recommendations on the protection of animals used for scientific purposes, Directive 2010/63 EU of the European Parliament and of the Council. The protocol was approved by the Italian Ministry of Health.

## Author Contributions

CTB, CU, CB, GP, and MMD contributed conception and design of the study. CU, DD, and FF performed the experiments. CTB, CU, and DD wrote the first draft of the manuscript. BO, GP, CB, and MMD provided key reagents and expertise. All authors contributed to manuscript revision, read, and approved the submitted version.

### Conflict of Interest Statement

The authors declare that the research was conducted in the absence of any commercial or financial relationships that could be construed as a potential conflict of interest.
