# Supplementary material for: A T Cell Suppressive Circuitry Mediated by CD39 and Regulated by ShcC/Rai Is Induced in Astrocytes by Encephalitogenic T Cells
Source: Front Immunol. 2019 May 10;10:1041. doi: 10.3389/fimmu.2019.01041 (PMC6524536; doi:10.3389/fimmu.2019.01041)
Supplement: Supplementary file 1 [file Data_Sheet_1.pdf]

A T cell suppressive circuitry mediated by CD39 and regulated by ShcC/Rai is induced in astrocytes by encephalitogenic T cells

Cristina Ulivieri[1*], Domiziana De Tommaso[1], Francesca Finetti[1], Barbara Ortensi[2,3], Giuliana Pelicci[2,3], Mario Milco D'Elios[4], Clara Ballerini[4], Cosima T Baldari[1*]

SUPPLEMENTARY MATERIAL
Supplemental information includes, three figures, one table and supplemental experimental procedures.

SUPPLEMENTARY FIGURES

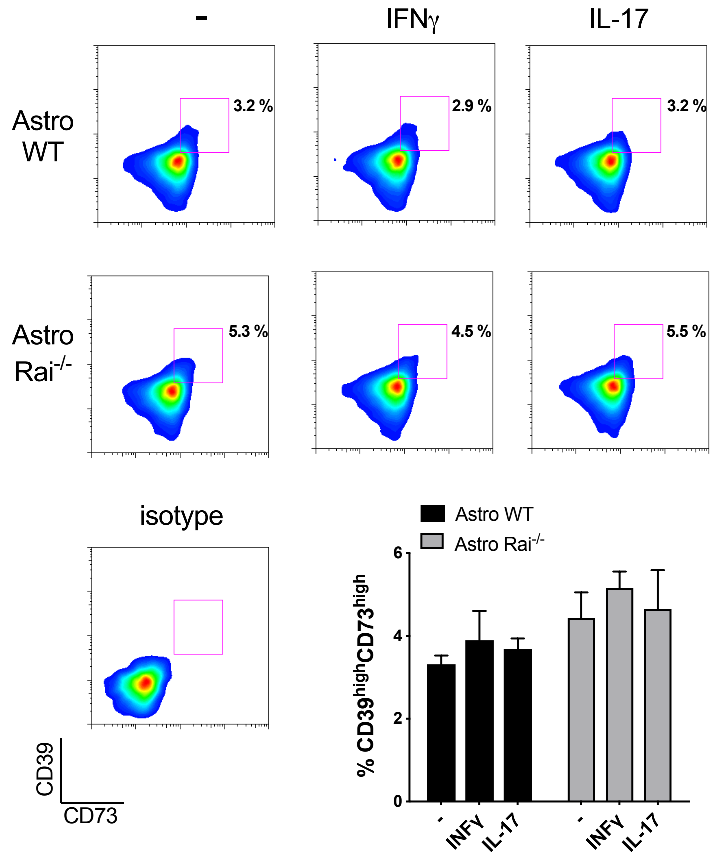

Supplementary Figure 1. Rai does not affect CD39 and CD73 expression in astrocytes following short-term stimulation with IL-17 or IFNγ. Flow cytometric analysis of CD73 and CD39 expression on $Rai^{+/+}$ (Astro WT) and $Rai^{-/-}$ (Astro $Rai^{-/-}$) astrocytes stimulated for 5 h with IFNγ (10 ng/ml), IL-17 (50 ng/ml) or left untreated (-) (mean % ± SD $CD39^{high}CD73^{high}$ astrocytes; n=3). Representative dots plot are shown.

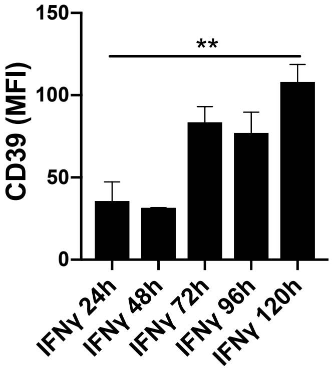

Supplementary Figure 2. Time course analysis of CD39 surface upregulation in WT astrocytes by flow cytometry. Data are presented as mean ± SD of mean fluorescence intensity (MFI). One-way ANOVA, multiple comparison (n>3), **p=0.0042 (IFNγ 24h versus IFNγ 120h).

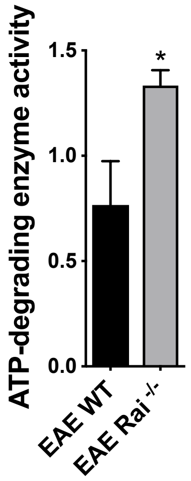

Supplementary Figure 3. Rai dampens extracellular ATP-degrading enzyme activity in glial cells of EAE mice. Quantification of enzymatic activities of extracellular ATP-degrading enzymes in adherent glial cells isolated from the spinal cord of $Rai^{+/+}$ EAE mice (EAE WT) and $Rai^{-/-}$ EAE mice (EAE $Rai^{-/-}$). Total glial cells were plated overnight in the absence of poly L-lysine. Adherent cells were washed and incubated with 1 mM ATP. Free phosphate production was measured as in Fig. 1D. Data are presented as mean fold change ± SD, with activity of cells from control mice taken as 1 (Mann-Whitney test; *p<0.05; 3 $Rai^{+/+}$ and 3 $Rai^{-/-}$ mice).

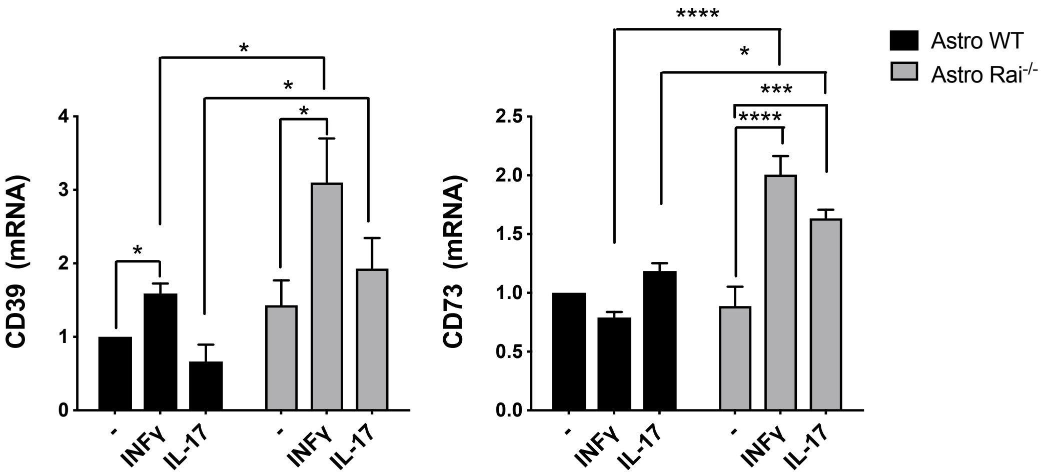

Supplementary Figure 4. Rai dampens IFNγ-dependent CD39 and CD73 mRNA expression. Real-Time PCR analysis of CD39 and CD73 mRNA expression in WT (Astro WT) and $Rai^{-/-}$ (Astro $Rai^{-/-}$) astrocytes untreated (-) or treated for 24 h with IL-17 (50 ng/ml) or IFNγ (10 ng/ml). Data from three independent experiments, each carried out on the pooled astrocytes from at least 5 $Rai^{+/+}$ or 5 $Rai^{-/-}$ mice, are presented as mean value ± SD, with the untreated sample of wild type astrocytes in each experiment taken as 1. 2-way ANOVA; **** p0.0001, ***p=0.0002, *p<0.05).

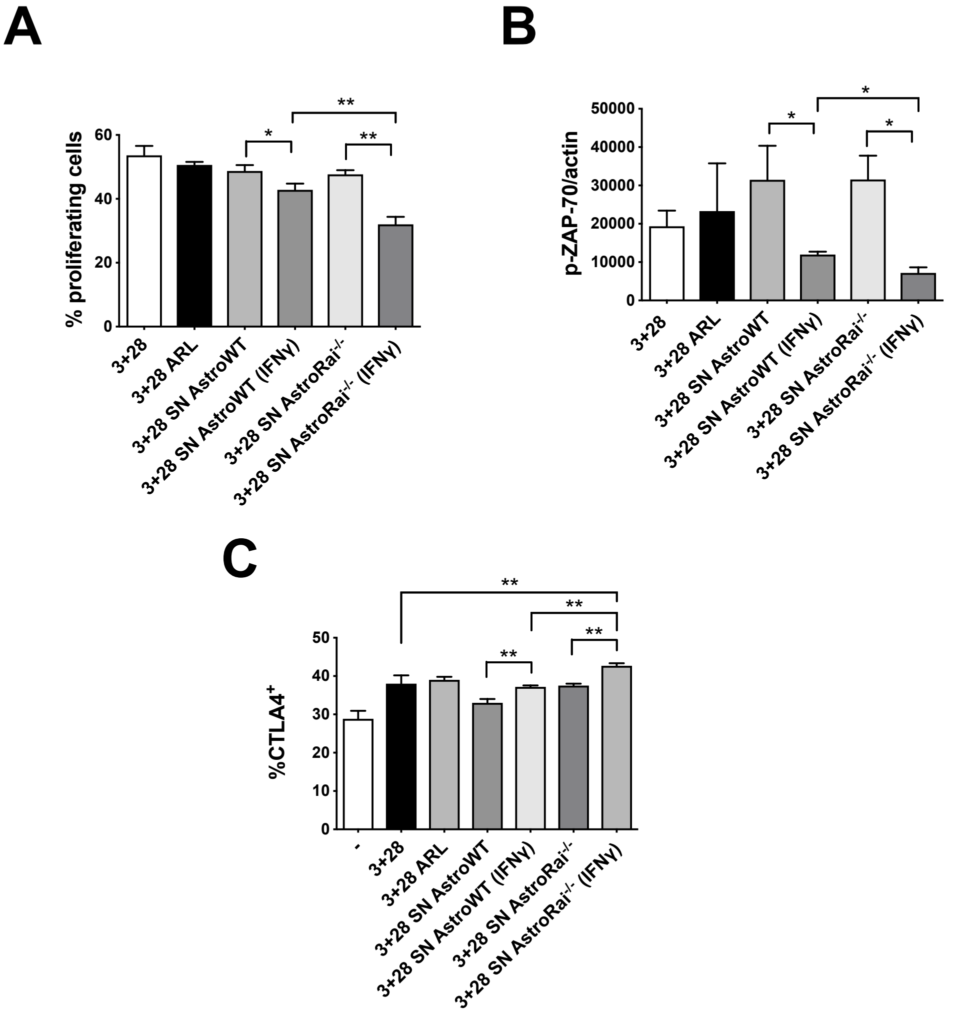

Supplementary Figure 5. Supernatants from IFN γ-treated astrocytes promote CTLA-4 expression and inhibit T cell activation and proliferation compared to supernatants from untreated astrocytes. (A) Flow cytometric analysis of CFSE-labeled splenic mouse cells from wild-type mice stimulated for 72 h with anti-CD3/CD28 antibodies (3+28) in combination with either supernatants from untreated Rai$^{+/+}$(SN AstroWT) and Rai$^{-/-}$ (SN AstroRai$^{-/-}$) astrocytes, or IFNγ-treated Rai$^{+/+}$ (SN AstroWT IFNγ) and Rai$^{-/-}$ (SN AstroRai$^{-/-}$ IFNγ) astrocytes. (B) Immunoblot analysis of ZAP-70 phosphorylation in postnuclear supernatants of splenocytes from wild-type mice stimulated for 5 min with anti-CD3/CD28 antibodies (3+28) in combination with either supernatants from untreated Rai$^{+/+}$(SN AstroWT) and Rai$^{-/-}$ (SN AstroRai$^{-/-}$) astrocytes, or IFNγ-treated Rai$^{+/+}$ (SN AstroWT IFNγ) and Rai$^{-/-}$ (SN AstroRai$^{-/-}$ IFNγ) astrocytes. (C) Flow cytometric analysis of the frequency of CTLA-4 positive cells among splenic mouse T cells treated as in A. Data obtained from cells stimulated with CD3/CD28 antibodies (3+28) in the presence or absence of ARL67156 (100 μM) (ARL) are also shown. Data are presented as mean value ± SD of the percentage of CTLA-4 positive cells or CFSE^{low} (proliferating) cells (n=4) and of the levels of phosphorylated ZAP-70 relative to actin (n=3). Mann-Whitney test, **$p < 0.01$, *$p < 0.05$.

SUPPLEMENTARY TABLE

Table 1. Related to Experimental procedures. Mouse primer sequences

ID	FWD	REV
Cd39	TCT GGG TGG AAC ATG TCA AA	ACC AGCCTC TGA GTC CTG AA
Emp1	GAGACACTGGCCAGAAAAGC	TAAAAGGCAAGGGAATGCAC
flCTLA4	CGCAGATTTATGTCATTGATCC	TTTTCACATAGACCCCTGTTGT
gapdh	AAC GAC CCC TTC ATT GAC	TCC ACG ACA TAC TCA GCA C
H2-D1	TCCGAGATTGTAAAGCGTGAAGA	ACAGGGCAGTGCAGGGATAG
S100a10	CCTCTGGCTGTGGACAAAAT	CTGCTCACAAGAAGCAGTGG
sCTLA4	CGCAGATTTATGTCATTGCTAA	TCATAAACGGCCTTTCAGTT
Serping	ACAGCCCCCTCTGAATTCTT	GGATGCTCTCCAAGTTGCTC

SUPPLEMENTARY METHODS

Generation of MOG-specific T cell lines

To generate $MOG_{35–55}$ specific T cells, female C57BL/6J mice were immunized with CFA and $MOG_{35–55}$. Seven days post-immunization, splenocytes and lymph nodes were harvested and expanded with 50 µg/ml MOG_{35-55} and 20 U/ml IL-2 in RPMI1640 with 10% BCS and penicillin, for 7 days at 37°C. Cells ($1x10^6$ cells/ml) were re-stimulated in the presence of $0.2x10^6$ cells/ml autologous bone marrow-derived dendritic cells, 50 µg/ml MOG_{35-55} and 20 U/ml IL-2, for 7 days. Cells underwent 2 rounds of stimulation before being stored at -80°C in 10% DMSO containing 90% FCS. Before usage cells were thawed and restimulated for 7 days with $0.2x10^6$ cells/ml DC, 50 µg/ml MOG_{35-55} and 20 U/ml IL-2. IL-17, IFNγ, GM-CSF and TNFα cytokine production was assessed by flow cytometry. Briefly, cells were stimulated with PMA (50 ng/ml) and ionomycin (1 µg/ml) in the presence of Brefeldin A for 4 h, after which GM-CSF-, TNFα, IFNγ- or IL-17a- producing MOG-T cells were analyzed by intracellular staining.

Isolation of glial cells from EAE mice

Spinal cords were isolated from EAE mice at the peak of disease (15 days postimmunization) and digested with 2.5 mg/ml collagenase D (Roche) and 1 mg/ml DNase I (Sigma-Aldrich) at 37°C for 45 min. Homogenates were passed through a 70- µm cell strainer. Cells were seeded on 48-well plates and incubated for 16 h for phosphate measurement.

Primary astrocyte culture and treatments

Primary astrocyte cultures were prepared from newborn mice as described (Colombo et al., 2012). Briefly, after removing the meninges, cerebral cortices were collected and dissociated using the Neural Tissue Dissociation kit (T) (Miltenyi Biotec, Bergisch Gladbach, Germany). Cells were plated on poly-L-lysine (10 µg/ml) coated T75-cm^2 flasks in DMEM supplemented with 10% bovine calf serum (BCS) and 20 U/mL penicillin. After 7 days, supernatants containing microglia were eliminated by shaking of the flask and adherent cells were trypsinized and replated. Cells that underwent 2 passages were used as astrocytes. The purity of astrocytes was ≥ 95% as assessed by immunostaining with Alexafluor488-conjugated anti-GFAP antibodies (Clone GA5, eBioscence).

For ATP,adenosine and phosphate measurements, $5x10^4$ astrocytes were left untreated or treated with IFNγ (10 ng/ml) or IL-17 (50 ng/ml) in serum-free DMEM for 5 h. Alternatively, primary astrocytes (2 $x10^4$ cells/ well in 96-well plates) were incubated with IFNγ or IL-17 in complete medium for 5 or 120 h for the assessment of CD39 and CD73 surface expression by flow cytometry. For the treatment with conditioned media from MOG-T cells, the culture medium was replaced with the culture supernatants from IL-2-stimulated MOG T cells in the presence or absence of a neutralizing anti-IFNγ mAb (e Bioscence). Alternatively, MOG T cells depleted of their culture supernatant and resuspended in fresh medium were added to astrocytes (ratio astrocytes: T cells 1:2) as such or previously pulsed with MOG_{35-55} (50 µg/ml) for 2 h. Cells were harvested at 120 h for FACS analysis. For qRT-PCR analysis of CD39 expression astrocytes ($5x10^4$ cells/sample) were left untreated or treated with IFNγ (10 ng/ml) or IL-17 (50 ng/ml) in complete medium for 24 h. For immunoprecipitation ($20x10^6$ cells/sample) were treated with IFNγ (10 ng/ml) for 15 min at 37°C.

Splenocytes, $CD4^+$ T cell purification and treatments

For proliferation assays and CTLA-4 expression cells ($5x10^6$ cells/sample) were treated with immobilized anti-CD3 (2 μg/ml; eBiosciences) and anti-CD28 (2 μg/ml; eBiosciences) mAb for 72 h, alone or in combination with either the nonhydrolyzable adenosine analog NECA (10 μM) (Sigma-Aldrich) or supernatants from IFNγ-treated $Rai^{-/-}$ or $Rai^{+/+}$ astrocytes, in presence or absence of the ectonucleotidase inhibitor ARL67156 (100 μM) (Sigma-Aldrich). For analysis of ZAP-70 activation splenocytes were either left untreated or pre-treated with supernatants from IFNγ-treated $Rai^{-/-}$ or $Rai^{+/+}$ astrocytes in the presence or absence of ARL67156 (100 μM) for 1 h at 37°C. T cells were activated for 5 min at 37°C by adding soluble anti-CD3 mAb and anti-CD28 mAb in presence or absence of 10 μM NECA. To assay CREB activation and PKA activity, $CD4^+$ T cells were either left untreated or treated with NECA (10 μM) or with supernatants from IFNγ-treated $Rai^{-/-}$ or $Rai^{+/+}$ astrocytes for 5 min at 37°C.

eATP, adenosine and ectonucleotidase activity measurements
For ATP measurements, astrocytes were incubated with serum-free medium containing IFNγ (10 ng/ml) or IL-17 (50 ng/ml) or medium for 5h. Cells supernatants were collected and cells were lysed in in 0.02% SDS in phosphate-buffered saline (PBS) for protein content determination. To measure total ATP levels, untreated cells were lysed in Lysis buffer (20 mM Tris-HCl (pH 8), 150 mM NaCl, 1% v/v Triton X-100) containing Protease Inhibitor Cocktail Set III (Calbiochem). Each sample was run in triplicate. For determination of nucleotide hydrolysis astrocytes or adherent glial cells obtained from the spinal cord of EAE mice were washed 3 times in Hepes buffer (2mM $CaCl_2 \times 2H_2O$, 120 mM NaCl, 5 mM KCl, 10 mM glucose, 20 mM Hepes, pH 7.4). Cells were then incubated in the same buffer with 1mM ATP for 30 min at 37°C. Reactions were stopped with trichloroacetic acid (final concentration 5%) and immediately placed on ice. Supernatants were collected and free phosphate was measured using the Malachite Green Phosphate Assay Kit (POMG-25H) (BioAssay Systems) at 620 nm on a microplate reader, according to the manufacturer's protocol. Remaining cells were lysed in 0.02% SDS in phosphate-buffered saline (PBS) and protein concentration were measured using the bicinchoninic acid (BCA) assay (Thermo Fisher Scientific). Specific activity was calculated using a calibration curve and expressed as nmol Pi released/mg protein/min.
To measure adenosine production culture supernatants of astrocytes were replaced by Hank's solution containing 1mM ATP and incubated for 60 min at 37°C. Supernatants were collected and adenosine concentration was measured using a fluorometric assay (Adenosine Assay Kit; Cell Biolabs, INC.) and a Fluorometer (TECAN) according to the manufacturer's instructions. Each sample was run in triplicate. Remaining cells were lysed in 0.02% SDS in phosphate-buffered saline (PBS) and protein concentration were measured using the BCA assay.

Cell lysis, Immunoprecipitations and immunoblots
Cells were lysed in 1% (v/v) Triton X-100 in 20 mM Tris-HCl (pH 8), 150 mM NaCl in the presence of Protease Inhibitor Cocktail Set III (Calbiochem) and 0.2 mg Na orthovanadate/ml. Postnuclear supernatants were resolved by SDS-PAGE and transferred to nitrocellulose. Alternatively, postnuclear supernatants were immunoprecipitated using RanBPM polyclonal antibody (Proteintech) and protein A Sepharose (GE Healthcare). Immunoblots were carried out using peroxidase-labeled secondary antibodies (GE Healthcare) and a chemiluminescence detection kit (Bio-rad Laboratories Inc, Milan, Italy). Immunoblots were scanned and quantitated using ImageJ software. The following primary Abs were used: anti-ShcC mAb (BD Biosciences), anti-RanBPM (A5) mAb (Santa Cruz Biotechnology) and anti-CD39 polyclonal antibody (Proteintech), anti-phospho-ZAP-70 (Y^{319}), anti-phospho-CREB (Ser^{133}) and anti-phospho-(Ser/Thr)-PKA substrates, purchased

from Cell Signaling Technology (CST). Antibodies against Actin (Millipore) or β-Tubulin (D2N5G) (CST) were used as loading controls.

Proliferation assays

Proliferation was measured by flow cytometry using the CFSE dilution method. Briefly, splenocytes were resuspended at $20x10^6$/ml in PBS and stained with 10 μM CFSE (Molecular Probes, Thermo Fisher Scientific) for 8 min at room temperature. Cells were subsequently washed twice in RPMI-10% BCS, resuspended at $5x10^6$/ml and treated with immobilized anti-CD3 (2 μg/ml; eBiosciences) and anti-CD28 (2 μg/ml; eBiosciences) mAb for 72 h, alone or in combination with either the nonhydrolyzable adenosine analog NECA (10 μM) (Sigma-Aldrich) or supernatants from IFNγ-treated $Rai^{-/-}$ or $Rai^{+/+}$ astrocytes, in presence or absence of the ectonucleotidase inhibitor ARL67156 (100 μM) (Sigma-Aldrich).

RNA purification and RT-qPCR

Total RNA was extracted from $1\text{-}5x10^6$ cells using the RNeasy Plus Mini Kit (Quiagen) according to the manufacturer's instructions. First-strand cDNAs were generated using the iScript™ cDNA Synthesis Kit (Bio-Rad). Real-time PCR was performed using the SsoFast™ EvaGreen® supermix kit (BIO-RAD). The cDNA fragments corresponding to mouse CD39, H2-D1, Serping, Emp1, S100a10 and the soluble and full-length isoforms of CTLA-4, were amplified using specific pairs of primers (Table 1). All samples were run in duplicate on 96-well optical PCR plates (Sarstedt AG). Gene expression data were normalized to the expression of the housekeeping gene GAPDH.
